# The use of bones as tools in Late Lower Paleolithic of Central Italy

**DOI:** 10.1038/s41598-024-62612-z

**Published:** 2024-05-22

**Authors:** Flavia Marinelli, Marie-Helénè Moncel, Cristina Lemorini

**Affiliations:** 1 Institute of Archaeology, Tel Aviv University, 69978 Tel Aviv, Israel; 2 UMR 7194 HNHP, CNRS-MNHN-UPVD,, Department Homme et Environnement, Muséum National d’Histoire Naturelle Paris, Paris, France; 3 Classic Department, LTFAPA Laboratory, “Sapienza” University of Rome, Rome, Italy

**Keywords:** Use-wear, Bone tools, Bone flakes, Lower Paleolithic, Experimental protocol, Italian Peninsula, Anthropology, Archaeology, Cultural evolution, Palaeontology

## Abstract

The Latium area in Italy has yielded rich evidence of Lower Paleolithic sites with both faunal remains, artefacts, and human fossil remains, such as the Ceprano human skull. Many are the sites where lithic industry has been found in association with bone industry. Medium and large animals were a key resource because they provided an enormous amount of meat and fat. However, they were extensively exploited for their bones, rich in marrow, and as raw material for tool production. Bone tools are so far few documented for early period of time and especially for the Middle Pleistocene in Western Europe. We report here evidence of bone tools and their efficiency of use for hominin groups living in the Frosinone-Ceprano basin during the MIS 11/10, a key period which records behavioral innovations and onset of the Neanderthal behaviors. In three sites, Isoletta, Colle Avarone and Selvotta, several bone tools and bone flakes have been discovered (MIS 11/10). They were associated to stone artefacts part of the hominins tool-kit. Technological and use-wear analyses conducted on these bone industries, dated between 410 and 430 ka, yield relevant results to understand the effectiveness of the bones tools found associated with lithic series, including handaxes.

## Introduction

Human–large mammal interactions occurred at many African, European, and Asian sites. In the Lower Palaeolithic, large animals were extensively exploited for their meat, fat, and bone marrow—high-energy nutritional sources—allowing hominins to have a high caloric intake^1–6^, and enabling them to survive over thousands of years.

Furthermore, bones served as raw materials for tool manufacturing, a practice dating back millions of years^7,8^. Some authors also suggest that bones were used for cosmological and ontological purposes in addition to their use as tools^4,9–14^.

Studies carried out by researchers in various parts of the world have revealed the importance of bone industry in the processing of animal carcasses and the removal of materials such as meat, tubers and termites, demonstrating the importance and effectiveness of bone tools in very ancient contexts^15–17^.

The use of bones for tool-making demonstrates the high cognitive abilities of hominins, who were able to distinguish the properties of raw materials and their potential applications.

During the middle Pleistocene, bone tools gained prominence, as evidenced by artefacts found at sites such as Marathousa (Greece), I and Schöningen (Spear Horizon) (Germany)^18,19^.

Bone flakes resulting from marrow extraction differ from the bone flakes of other large animals, such as elephants. It appears that hominins intentionally produced large bone flakes to realise the production of bone tools, including bifaces^10^.

While bone retouching and specific punctual bone industries are documented in the European Middle Palaeolithic, little evidence exists for the earliest periods, particularly towards the end of the Middle Pleistocene^20^. In Italy, evidence of bone artefacts crafted from large bone fragments is evidenced in the Lower Palaeolithic, particularly between MIS 13 and MIS 9, a key period recording the onset of Neanderthal activity^4,21,22^.

Here, we present the first description of bone tools acquired from three archaeological localities in the Frosinone-Ceprano (southern Latium) basin. Use-wear analysis was employed investigate the use of these tools. These sites are Isoletta, Colle Avarone, and Selvotta, and the material is stored in the Pofi Museum.

Identification of the specific use of these bones allowed us to emphasise the concept of human–large animal interaction and, in particular, to highlight the importance that these early bone tools had for the success of precise activities essential to the life of Lower Palaeolithic hominins.

## The archeological sites of Isoletta, Colle Avarone and Selvotta

The sites are located in the lacustrine formations of the Frosinone-Ceprano basin. Similar stratigraphic contexts were evidenced by I. Biddittu in 1974 on the Campogrande, Colle Avarone and Selvotta outcrops, which are mainly composed of gravels, sands and sandy clays.

Due to the numerous geochronological data obtained through the ^40^Ar/^39^Ar method on volcanic minerals and the ESR/U series, it was possible to chronologically frame these lower Palaeolithic sites, dating them between 410 and 430 ka^23^.

### Isoletta

The archaeological site of Isoletta, which was discovered in 1970, is located a few kilometres SE of Ceprano city at the confluence of the Sacco and Liri Rivers. The stratigraphic sequence of the Isoletta site is characterised by alluvial deposits consisting of sand, gravel, and volcanic material^23^.

A survey carried out in 1998–99 during the construction of the high-speed railway revealed two archaeological levels at the site: the lower level, where rare lithic industry archaeological material from the Mode 1 industry was found, along with large mammals (elephants), birds, amphibians, and fish remains. An elephant tibia intentionally fractured by humans was found at this level^24,25^; the upper level (GA6Z) Acheulean industry in flint and limestone included bifaces, choppers, scrapers and denticulates^23,24^. The faunal remains included large animals such as *Elephas antiquus*, *Bos primigenius*, *Cervus elaphus*, and *Dama clactoniana*. Palaeontological remains found at level 4 are associated with the Galerian large mammal assemblage^26,27^.

### Colle Avarone

Colle Avarone was identified in 1973 by I. Biddittu. The site, identified in a former “pozzolana” quarry, is located in the lower Sacco River Valley a few kilometres from the confluence of the river with the Liri River. According to an assay carried out by I. Biddittu in 1975, an artefact of bone made from an elephant rib was found^23^.

In the main archaeological level, tools consisting of the Acheulean industry on bone, flint and limestone were found in fluvio-lacustrine deposits consisting of gravels and volcanic products. The artefacts include mainly bifaces and other artefacts, including flakes^28^.

The faunal assemblage consists mostly of large mammals (*Palaeoloxodon antiquus, Sus scrofa, Equus caballus, Bos primigenius, Cervus elaphus*), birds *Stercorarius longicaudatus, Anser brachyrhycus*) and turtles (*Testudo* sp.).

### Selvotta

The site, the data for which are still unpublished, is located near Colle Avarone. Both lithic and bone tools were recovered at this site. Italo Biddittu proposed a geological correlation between the archaeological level of Selvotta and the archaeological level of Colle Avarone (CA) and the archaeological levels CG 9 and CG 10 above the Campogrande localities. A single archaeological level was found, and most of the lithic and bone remains consist of surface collections. Among the finds, both lithic and bone artefacts were found. All these levels are characteristic fluvio-lacustrine deposits, with gravels and volcanic minerals, of the Lirino palaeolake that formed during the MIS 11/MIS 10 transition period^29,30^.

### Selected item and state of preservation

A total of 32 bone items were analysed: 23 from the Isoletta site (Frosinone), eight from Colle Avarone (Ceprano, Frosinone), and one from Selvotta Selvotta (Ceprano, Frosinone) (Supplementary Figs. 1–7).

Despite localised concretions and slight surface abrasions caused by a few random striations (Supplementary Fig. 8), the bones were exceptionally well preserved, which was confirmed through use-wear analysis.

Furthermore, the good state of preservation of these bone products and the very low degree of fragmentation led us to investigate possible use-related wear at the macro, meso- and microscales.

The selected bone tools were all unretouched except for two, one from the Isoletta and one from the Colle Avarone site. The tools mostly present a morphology with a pointed end used as the active area of the item, but by a smaller number of tools have an active edge localised in the mesial portion of the item.

## The experiments

A series of experiments were conducted to characterise the use and wear of replicas of bone fragments, which is pivotal for interpreting the analysed archaeological traces.

The experiments were carried out by five people who had experience in these activities.

**Figure 1:**
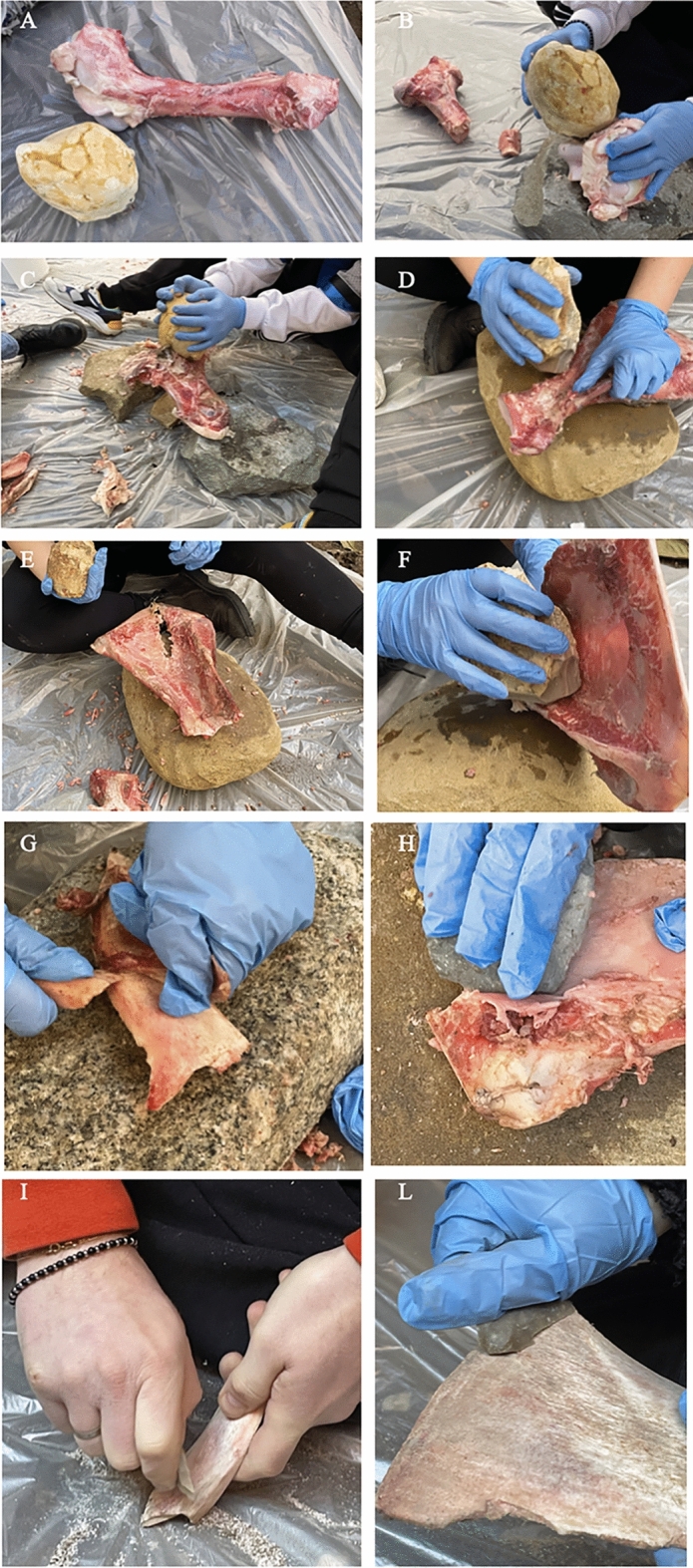
Experiments. (**A**–**F**) Tool breakage for the production of bone tools, placing it on an anvil and using a hammer stone. (**G**–**L**) Removal of meat residue from bones, using flint and bone flakes.

The first phase of experimentation consisted of making bone tools that were similar to the archaeological tools. Bovine bones, including the scapula, femur and ribs, were used. These bones underwent fracture via anvil percussion (Fig. 1A–F), followed by cleansing using three flint flakes with a fine-grained texture and two bone fragments (Fig. 1G–L) and were left to dry under ash.

The activities conducted included wood working (debarking), butchering, and soil excavation.

A distinct potentially functional area was identified on each tool for the intended activity, including sharp and straight edges. The active edge remained unmodified by retouch.

**Figure 2:**
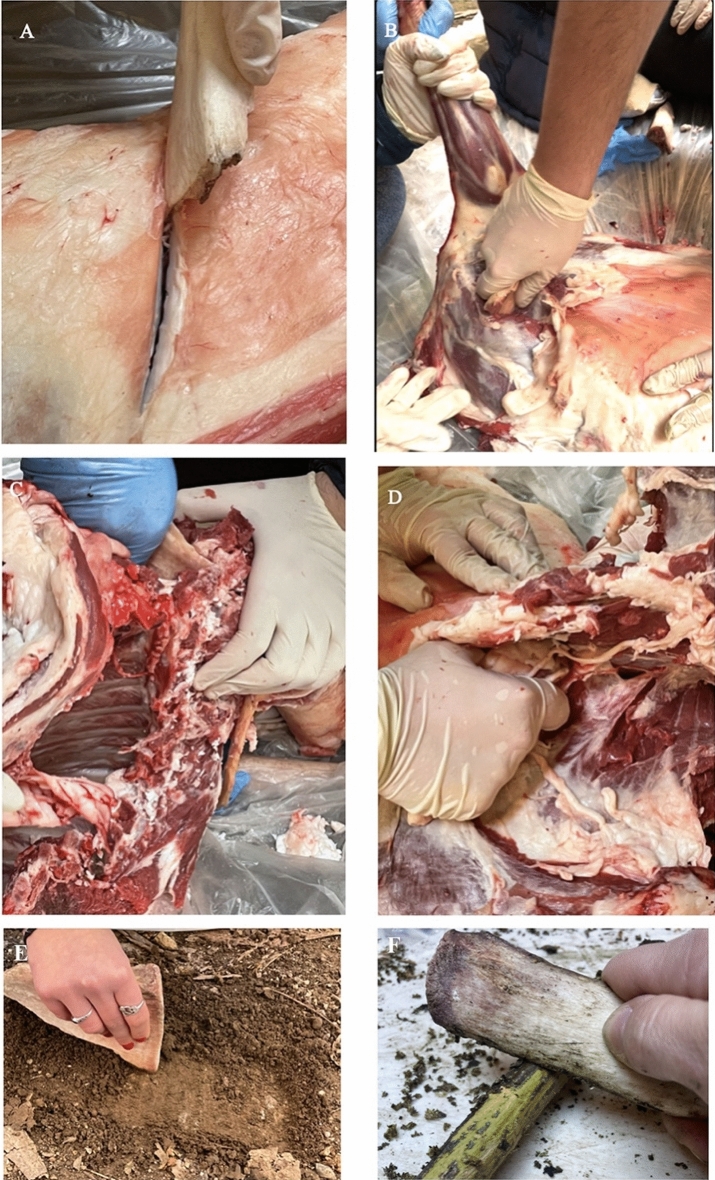
Experiments. (**A**) Butchering sheep: incision of the carcass using a bone flake; (**B**) Separating leg from the carcass; (**C**, **D**) Removing meat portion from the ribs using bone tools. (**E**) Soil digging using bone tool. (**F**) Scraping wood using bone tool.

Butchering was performed over 2 hours. The first step involved separating the legs of sheep from an adult sheep carcass (Fig. 2A,B) and subsequently removing the meat portion from the rib (Fig. 2C,D). The tool, possessing a pointed end, made it possible to carry out an incision and subsequently make the cut very quickly; the tendons and ligaments required more force and time.

Soil and wood experiments were easily carried out and were performed in one hour. Flatter and straight-edged bones, such as the scapula (for soil digging) and ribs (for debarking wood), were used for these activities (Fig. 2E,F).

During this experiment, it was observed that the tool edge tended to quickly round and lose its effectiveness, likely due to the friction generated by the processed material, which caused dulling of the active edge.

In the process of debarking wood, the mesial portion of the tool was identified as the active edge, as it exhibited better adherence to the surface being processed (Fig. 2F).

### Use-wear analysis

Use-wear analysis carried out on the experimental bone fragments showed similarities with the traces found on the archaeological sample, particularly in experiments involving butchering and digging activities.

**Figure 3:**
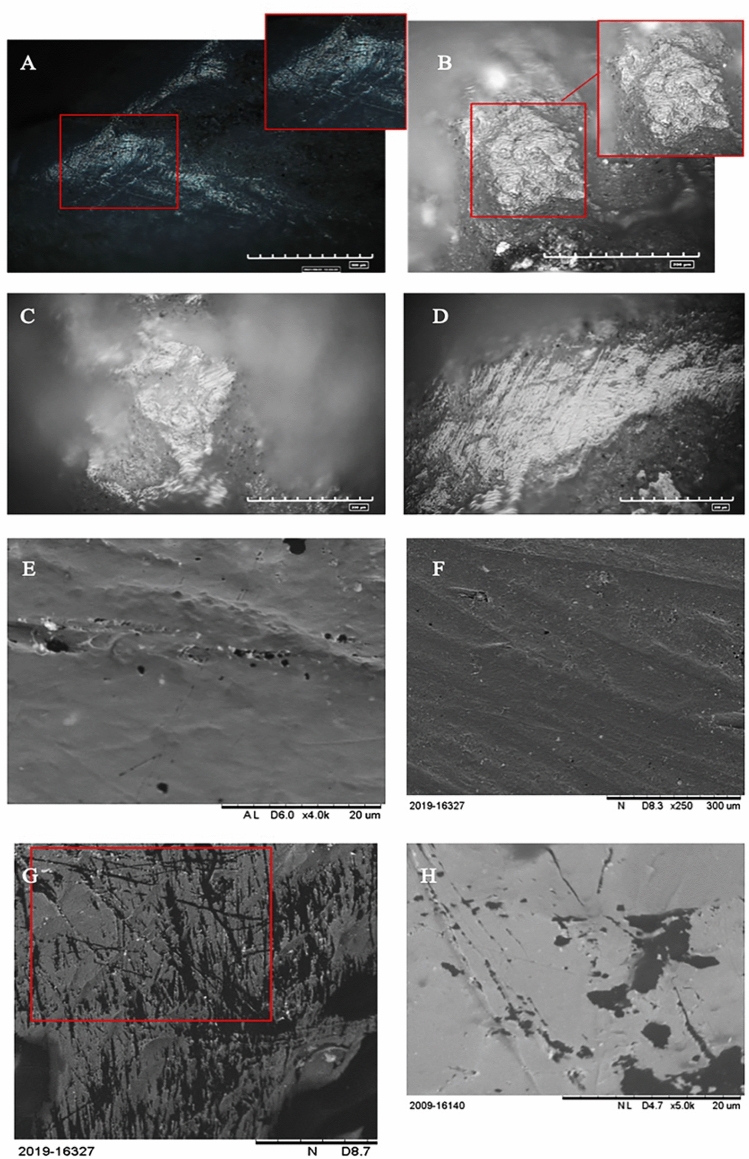
Polishes localised on archaeological and experimental items. (**A**) Butchering activities. Polish located on the active edge; (**B**) Isoletta #8618. Polish present on the active edge of the archaeological fragment analysed; (**C**) Colle Avarone #9367. Polish and striae concentrated on the active edge of the item; (**D**, **E**) Isoletta #13501. Polish and striae on the active edge; (**F**–**H**) Butchering activity. Morphological analysis of shallow, rounded striae.

In relation to butchering activity, Fig. 3 shows analogies between the experimental and archaeologically active edges. Microscopic analysis of the tool revealed overall rounding on the active edge employed for meat removal from the animal carcass.

Polishes were evident along the rounded edge of the tool, characterised by a smooth texture (Fig. 3A) similar to that of the archaeological active edge (Fig. 3B–D) and longitudinally shallow striae distributed longitudinally along the active edge that showed rounding (Fig. 3F–H). SEM analysis highlighted alternating degrees of rounding between poorly developed areas and areas with more pronounced rounding (Fig. 3F). Pronounced rounding correlated with an incision made into fattest area of carcasses, such as the epidermis. As hypothesised for the archaeological sample (Fig. 3D), the tool’s adherence to the fattier meat sections led to increased rounding of the striae. Conversely, softer and less fatty internal carcass areas resulted in moderate rounding.

**Figure 4:**
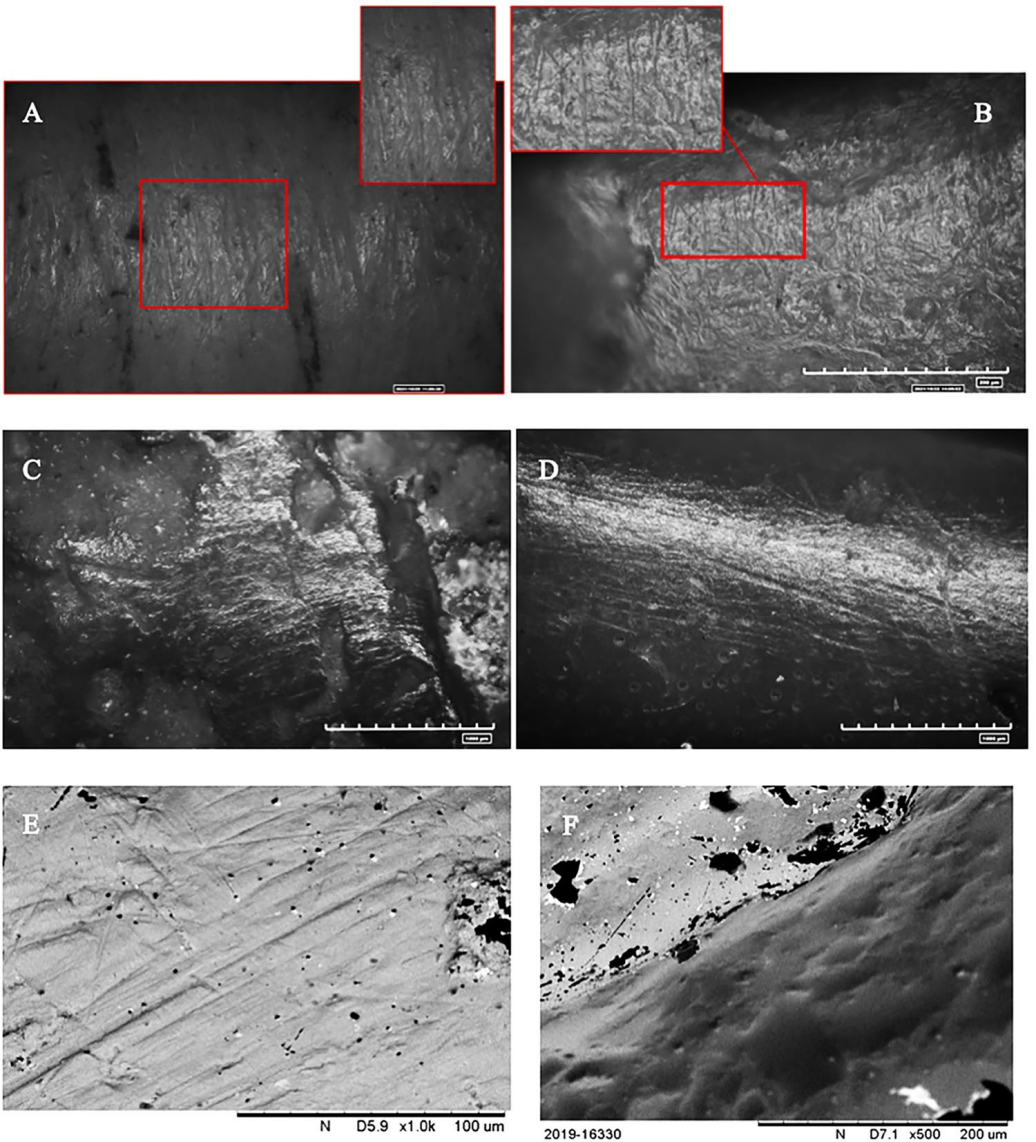
Stries and polishes localised on archaeological and experimental items. 4. Soil excavation activities. (**A**) Presence of striae along the active edge of the experimental item; (**B**) Isoletta #13027. Striae highlighted along the active edge of the archaeological item; (**C**) Isoletta #4169. Striae and polish concentrated on the edge of the tool; (**D**) Colle Avarone #8755. Striae and polish on the active edge of the tool. (**E**) Isoletta #4169. SEM morphological analysis of the striae present on the active edge. (**F**) Morphological analysis of the striae present on the experimental active edge.

A comparison of use-wear on the archaeological and experimental tools suggested that the majority of the analysed tools were employed in butchering activities. Furthermore, similarities were observed in the tools used in the soil excavation experiments (Fig. 4).

**Figure 5:**
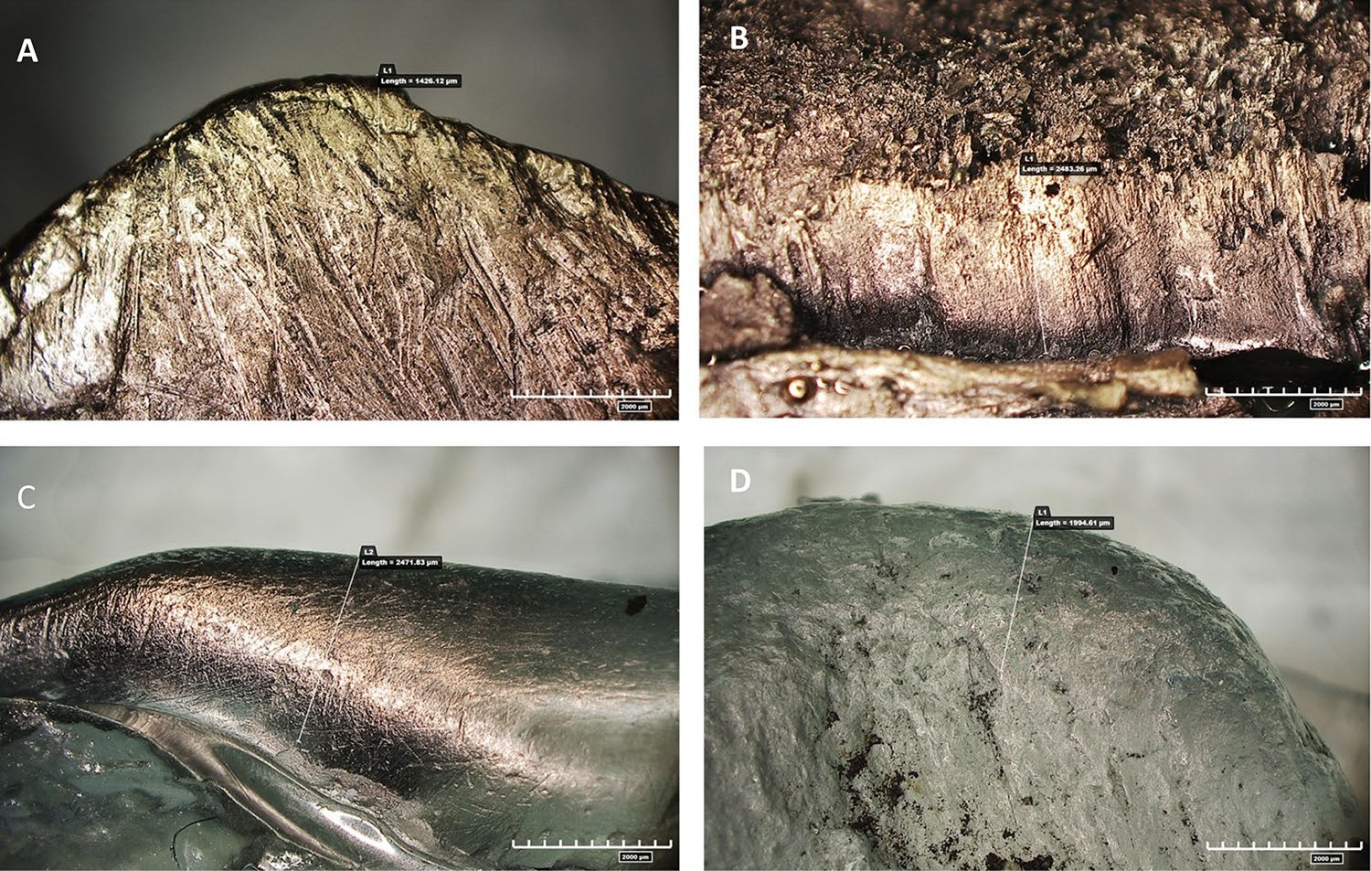
Differences between archaeological and experimental use-wear. (**A**) Rounding and stries of the active edge generated by butchering activity; (**B**) Rounding and stries of the active edge generated by digging soil; (**C**) Rounding and strie of the active edge of archaeological item generated by soft material; (**D**) Rounding and strie of the active edge of archaeological item generated by hard material.

The use-wear analysis of the soil excavation tools revealed pronounced rounding of the active edge, exceeding the rounding observed in the tools used for butchering (Fig. 5). The difference from meat processing was accentuated by the formation of deeper striae (Fig. 5C,D), attributable to the intense friction and granular nature of the soil, which consistently worn down the tool.

Microscopic analysis revealed the presence of polishes characterised by a rough-to-smooth texture, typical of contact with a resistant material such as soil, that showed strong similarities with the polishes of sample items #13027, #4169, and #8755 (Fig. 4B–D). SEM analysis revealed substantial rounding of the striae, confirming, through comparison with archaeological findings, the hypotheses regarding this type of activity (Fig. 4E,F).

## Results

### General considerations for sample and bone tool functions

Each analysed item was classified based on its morphological characteristics. These parameters (see Table 1) combined with the reference collection data enabled the interpretation of the use of these items.

**Table 1:** Characteristics of the analysed sample.

ID	Site	Type of alteration	Anthropic detachments	Pointed area	Rounding on the active edge	Natural rounding	Use-wear and striae	Edge angle of the active edge	Length of the item (mm)
M 4239	Isoletta	Slightly altered	No	Yes	Yes	No		25°	280
M4168	Isoletta	Slightly altered and presence of concretion	No	No	Yes	No		23°	130
M13032	Isoletta	Slightly altered and presence of concretion	No	Yes	Yes	No	Presence of microtraces and striae	22°	120
M13027	Isoletta	Slightly altered and presence of concretion	No	Slightly pointed	Yes	No	Presence of microtraces and striae	21°	100
M13034	Isoletta	Slightly altered	No	Slightly pointed	Yes	No	Presence of marks on the surface	21°	120
M13038	Isoletta	Altered and presence of concretion	No	Yes	Yes	No	Presence of microtraces and striae	25°	180
M4245	Isoletta	Altered	Yes (presence of detachment on the tip of the item)	Slightly pointed	Yes	Yes	Presence of microtraces and striae	19°	150
M13029	Isoletta	Altered	No	No	Yes	No	Presence of striae on the surface	21°	130
M13044	Isoletta	Altered	No	Yes	Yes	Yes	Present of pronunced striae	18°	230
M13046	Isoletta	Altered	No	No	Yes	No	Presence of microtraces	23°	120
M13045	Isoletta	Altered and presence of concretion	No	Yes	Yes	Yes		27°	170
M13036	Isoletta	Slightly altered and presence of concretion	No	Yes	No	No	Presence of microtraces and striae	24°	130
M10121	Isoletta	Slightly altered	Yes (Presence of the detachment in the opposite area of the active edge)	Yes	Yes	Yes	Presence of microtraces and striae	20°	170
M13037	Isoletta	Altered and presence of concretion	No	Yes	Yes	Yes	Presence of microtraces and striae	25°	130
M4168	Isoletta	Altered and presence of concretion	No	Yes	Yes	Yes		26°	150
M13501	Isoletta	Altered	No	Yes	Yes	Yes	Presence of striae	27°	260
M4169	Isoletta	Slightly altered	Yes (Presence of the detachment on the active edge of the item)	Yes	Yes	Yes		24°	400
M4166	Isoletta	Altered	Yes (Presence of the detachment on the active edge of the item)	Yes	Yes	Yes		26°	260
M13030	Isoletta	Altered	No	Yes	Yes	Yes	Presence of striae and bright spot	21°	160
M12965	Isoletta	Altered and presence of concretion	Yes (Presence of the detachment on the active edge and on the masial portion of the item)	Yes	Yes	Yes	Presence of microtraces and stirae	22°	170
M4243	Isoletta	Altered and presence of concretion	No	Yes	Yes	Yes	Presence of striae	25°	250
M4244	Isoletta	Altered and broken	No	No	Yes	Yes	Presence of microtraces and striae	26°	250
M4235	Isoletta	Altered and presence of concretion	No	Yes	Yes	No	Presence of microtraces and striae	27°	150
M9367	Colle Avarone	Altered	No	No	No	Yes	Presence of microtraces and striae	24°	80
M9369	Colle Avarone	Abraded	No	No	No	No		25°	90
M9366	Colle Avarone	Slightly abraded	No	No	Yes	Yes	Presence of microtraces and striae	28°	60
M8618	Colle Avarone	Slightly abraded	Yes (Presence of the detachment on the active edge of the item)	Yes	Yes	No	Presence of striae	33°	170
M8753	Colle Avarone	Slightly abraded	Yes (Presence of the detachment in the area opposite of the active edge)	Yes	Yes	Yes	Presence of striae	25°	170
M8755	Colle Avarone	Slightly abraded	No	No	Yes	Yes	Presence of microtraces and striae	26°	120
M8930	Colle Avarone	Abraded	No	No	No	Yes	Presence of macrotraces	25°	80
M8931	Colle Avarone	Abraded	No	Yes	Yes	No	Presence of macrotraces	24°	70
M10119	Selvotta	Slightly abraded	No	Yes	Yes	Yes	Presence of microtraces and striae	25°	250

**Figure 6:**
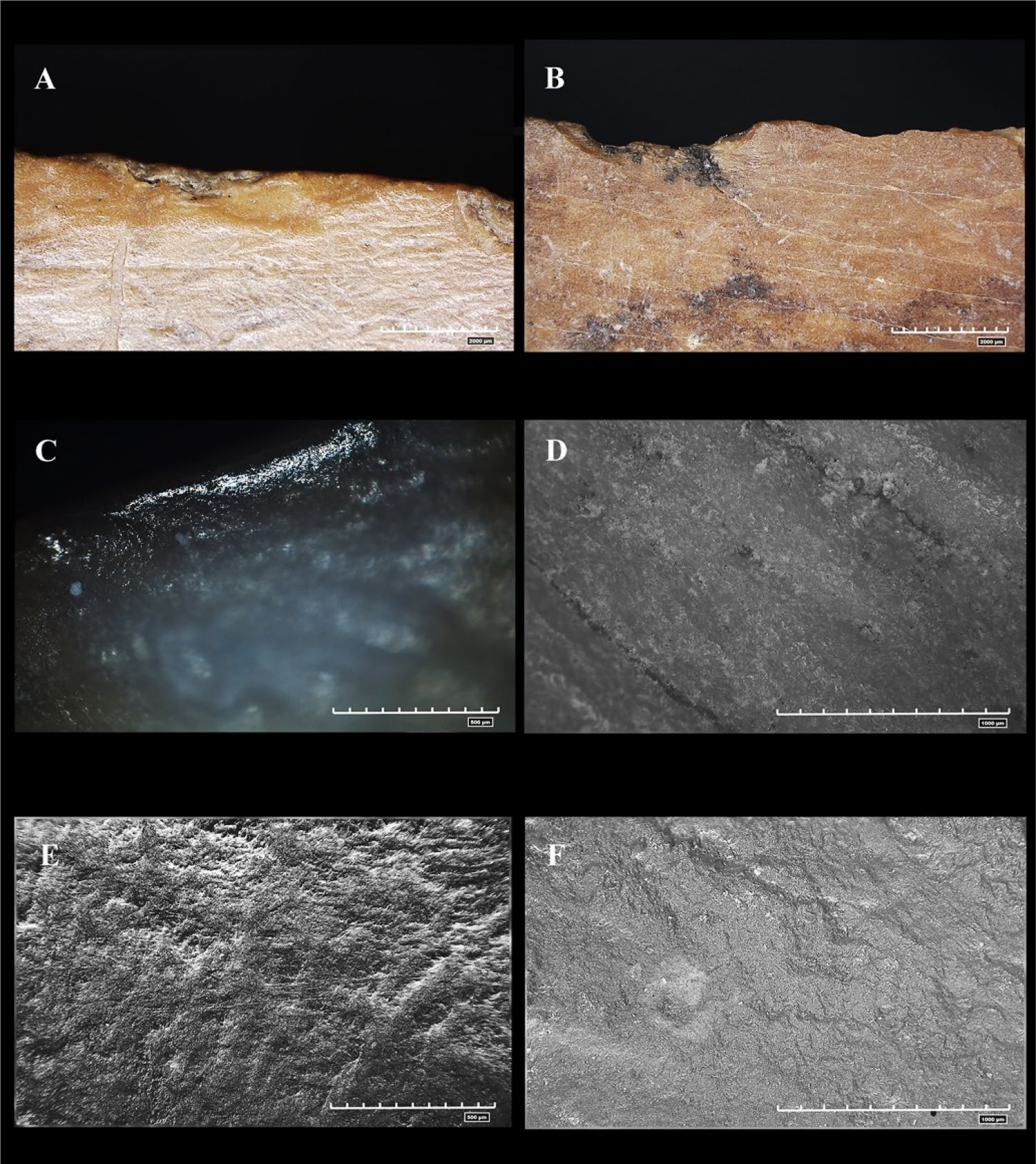
Differences between active edge and unused edge. (**A**) active edge used; (**B**) active edge unused; (**C**) Polish located on the active edge; (**D**) Surface with the absence of polish; (**E**) Presence of the striae on the item; (**F**) Absence of the striae on the item.

The following wear categories were established: edges with rounding resulting from bone versus unused edges; tool-generated structures versus natural bone structures; and use-related wear versus wear caused by post-depositional factors (Fig. 6).

This classification is established in the literature. Abrasion is caused by mineral particles carried by water and wind.

Unlike human-induced abrasion, general abrasion is not localised but spreads across the whole bone surface. The natural rounding of edges due to abrasion was distinguished from edges polished through human sharpening with abrasive materials^31–33^.

Bone modifications by hominins can arise from activities such as butchering and disarticulation^34^, resulting in the formation of striae (cutmarks, scrapemarks). These exhibit specific characteristics: striae from human activities are noted for being parallel, closely spaced, and narrow with short lines on bones.

Upon examination of the analysed sample, it was observed that some polishes and striae were concentrated at specific points, while others extended across the entire bone surface. The close, parallel, linear, and narrow striae were closely associated with the rounded edges. These differed from natural bone striae, which are generally more superficial and run along the entire bone surface.

For the Isoletta site, out of the 23 items examined, edge rounding, polishes and striae were observed on 18 items, while four items (four out of 23 items) showed only edge rounding. The traces on one item (one out of 23 items) were uninterpretable.

In the case of Colle Avarone, six out of seven items showed use-wear, while the use for one item was not interpretable (one out of seven items). At the Selvotta site, use-wear was analysed for only one item.

All the items show a recurrence in the morphological features and distribution of the diagnosed traces.

In general, bone fragments are characterised by very pronounced rounding of the distal, pointed edge of the tool (Supplementary Fig. S9), while in rare cases, rounding is present in the mesial portion of the active edge.

A specific example is the tool (#8618) from the Colle Avarone site. This tool exhibited considerable rounding of the active edge and revealed negative detachment resulting from tool retouching before activity (Supplementary Fig. S10). Further examination of these roundings was conducted using a metallographic microscope, digital microscope (Hirox) and scanning electron microscope (SEM), which confirmed the presence of striae and polishes.

### Striae and Polishes on the bone tools

The striae were distinguished based on their depth (shallow and deep), shape (straight and sinuous) and orientation (perpendicular or longitudinal to the active edge).

Their location was a crucial factor in interpreting use-wear, which involved three identified parameters: striae present on the outer edge, striae distant from the active edge, and the distribution of traces consistent with the direction of use.

Microscopic analysis also revealed a chaotic distribution of striae on the item surfaces. This distribution is associated with post-depositional events, e.g., ground particles might have caused abrasion, resulting in scratching on the bone surface (Supplementary Fig. S11).

SEM analysis highlighted the degree of rounding of the striae, which tended to be shallow in most cases, indicating potential processing of a low-resistance material.

Associated with these striae, microscopic analysis revealed the presence of low-shinning polishes, referred to as “matts,” mostly developed along the active edge or slightly shifted towards the surface. These polishes show a continuous trend from a rough to a smooth texture.

Concerning the Isoletta site, item #4245 shows a combination of striae, polishes and rounding at its edge. Numerous shallow striae scratching the polishes developed on the active edge with longitudinal and perpendicular orientations. The observed smooth texture of the polishes suggested the processing of a soft material. Detailed morphological analysis through SEM at high magnification revealed roundings on the striae, further supporting this interpretation (Supplementary Fig. S12).

Comparative analysis with the experimental traces allowed us to hypothesise that this tool was used to process materials of soft consistency, such as hides that required mixed modes of motion. Friction generated during contact between the tool and the material being processed likely caused the rounding of the striae. Item #4010, which displayed shallow longitudinal striae and associated smooth-textured polishes, indicated the processing of softer material (Supplementary Fig. S11).

SEM analysis revealed less pronounced rounding of the striae in this sample than of the other bone fragments, suggesting less friction, likely due to contact with a softer material, such as meat.

For item #13027, the rounding was located in the distal portion of the fragment, which is characterised by a pointed shape. Microscopic observation revealed rounded active edges with perpendicular striae exhibiting significant rounding, accompanied by polishes displaying a rough-to-smooth texture (Supplementary Fig. S13).

This type of trace allowed us to hypothesise that the tool was used for the processing of stronger material, such as soil, that generated more friction on the surface of the item, as evidenced by the degree and extent of rounding of the pointed area.

Similar traces were noted on bone items found at the Colle Avarone. Item #8618 shows highly localised and developed rounding of the active edge. Additionally, the edge displays small detachments overlapped with larger detachments, indicating knapping activity aimed at shaping the tool before use (Supplementary Fig. S10).

Microscopic analysis revealed polishes with a smooth texture, longitudinal orientation and shallow unidirectional striae, confirming the use of the item in the cutting of soft material.

Similar use-wear patterns were also identified at the Selvotta site. Bone fragment #10119 shows shallow striae with a transverse orientation characterised by pronounced rounding (Supplementary Fig. S14). The presence of these features, together with polishes with rough-to-smooth texture, suggests contact with a material that, while not highly resistant, generated enough friction to cause substantial rounding. Therefore, this fragment was likely used for processing soft materials, such as hides.

## Discussion

The use-wear analyses carried out on the Isoletta, Colle Avarone, and Selvotta sites demonstrated the involvement of bone tools in both butchering activities and ground excavation.

The polishes and the depth of the striae served as crucial parameters for distinguishing between the different types of materials processed.

Due to the polishing, it was possible to distinguish between soft materials and hard materials. However, the depth of the striae allowed for discerning the degree of abrasion caused by the processed material. In fact, as the table shows, softer materials such as meat tend to generate shallow striae on the tool, while tools exhibiting deeper striae are likely to be used for processing stronger materials (Tables 1 and 2).

**Table 2:** Parameters of the archaeological and experimental use-wear.

Id	Edge rounding	Polishes	Striaes	Striaes rounding	Interpretation
Isoletta					
# 4168	Yes	–	–	–	Indet
# 4239	Yes	–	–	–	Indet
# 4245	Yes	Smooth	Shallow-longitudinal and transversal orientation	High on the external portion of the striae	Working soft material (hide)
# 10121	Yes	–	Shallow-Longitudinal orientation	Medium on the external portion of the striae	Working soft material (probably meat)
# 13027	Yes	Rough-to-smooth	Deep-Transversal orinetation	High on the external portion of the striae	Working hard and abrasive material (ground)
# 13037	yes	smooth	Shallow longitudinal orientation	Medium on the external portion of the striae	Working soft material (meat)
# 13038	Yes	smooth	Shallow longitudinal orientation	High on the external portion of the striae	Working soft material (hide)
# 13044	Yes	Rough-to-smooth	Deep-transversal orientation	High on the external portion of the striae	Working hard and abrasive material (ground)
# 13046	Yes	–	Deep-longitudinal orientation	High on the external portion of the striae	Working soft material (hide)
# 13501	Yes	Smooth	Shallow- longitudinal orientation	Medium on the external portion on the striae	Working soft material (meat)
# 4010	Yes	Smooth	Shallow- longitudinal orientation	Medium on the portion of the striae	Working soft material (probably meat)
# 4166	Yes	–	–	–	–
# 4169	Yes	Rough-to-smooth	Deep-transversal orientation	High on the portion of the striae	Working hard and abrasive material (ground)
# 4235	Yes	Rough-to-smooth	Deep-longitudinal orientation	High on the portion of the striae	Working hard and abrasive material (ground)
# 4244	Yes	–	–	–	–
# 12965	Yes	Smooth	Shallow-londitudinal orientation	Medium on the external portion of the striae	Working soft material (meat)
# 13029	yes	–	–	–	–
# 13030	Yes	Altered-bright spots	Shallow-longitudinal orientation	Medium on the external portion of the striae	Working soft material (probably meat)
# 13032	Yes	–	Shallow-longitudinal orientation	High on the external portion of the striae	Working soft material (hide)
# 13034	Yes	Rough-to-smooth	Deep-longitudianl orientation	High on the external portion of the striae	Working hard and abrasive material (ground)
# 13036	Yes	Alterated-bright spots	–	–	–
# 13045	Yes	–	–	–	–
Colle Avarone					
# 8618	Yes	Smooth	Shallow-longitudinal orientation	Medium on the external portion of the striae	Working soft material (meat)
# 8753	Yes	–	Shallow- longitudinal orientation	Medium on the external portion of the striae	Working soft material (probably meat)
# 8930	Yes	–	–	–	–
# 8931	Yes	–	–	–	–
# 9366	Yes	–	Shallow- longitudinal orientation	Medium on the external portion of the striae	Working soft material (probably meat)
# 9367	Yes	Smooth	Shallow-longitudinal orientation	Medium on the external portion of the striae	Working soft material (probably meat)
# 9369	–	–	–	–	–
Selvotta					
# 10,119	Yes	Rough-to-Smooth	Shallow-longitudinal orientation	High on the external portion of the striae	Working soft material (hide)

Experiments					
Id	Activity	Edge rounding	Microtraces	Striaes	Striaes rounding
#3 fragment of femour	Cutting meat	Yes	Smooth	Shallow-longitudinal orientation	Medium on the external portion of the striae
#3 fragment of femour	Engraving meat	Yes	Smooth-rough to smooth	Shallow-longitudinal orientation	High on the external portion of the striae
#8 fragment of scapula	Working ground	Yes	Rough to smooth	Deep-transversal motion	High on the external portion of the striae
#5 fragment of scapula	Working ground	Yes	Rough to smooth	Deep-transversal motion	High on the external portion of the striae
#7 rib	Working ground	Yes	Rough to smooth	Deep-trasversal motion	High on the external portion of the striae

The degree of rounding of these striae was an important factor in interpreting the use of the artefacts, notably in hiding and soil processing. Experimentation highlighted a difference in the rounding of the striae generated by meat and hide processing. Striae were less rounded in tools used for meat processing due to the lower fat content of the material, while hide processing resulted in more rounded striae. This discrepancy may be attributed to the viscosity of hide, which causes more friction against the tool.

The presence of microtraces and the depth of the striae were equally significant in distinguishing tools used for hide processing from those used for soil working. As illustrated in the summary table (Table 2), the rounding of the striae appears identical in both cases, as extreme rounding of the active edge was generated by friction with the worked material.

However, the disparity between the two types of material is evident in the nature of the polishes and the depth of the striae. In hide processing, the striae tend to be shallow, with the polishes exhibiting a smooth texture. In contrast, soil processing yields deeper striae with rough-to-smooth textured polishing. This distinction arises from differences in friction and the textures of the processed material. Soft and more viscous materials are generally less granular than materials such as soil, which contain numerous inclusions such as gravel, sand and plant residues.

Analysis of the items from the three Southern Latium (Italy) sites indicated that not all tools showed signs of intentional knapping. In fact, some tools have no signs of shaping by knapping. The edge was considered to be effective immediately after detachment from the bones. This hypothesis was confirmed through experimental data, suggesting that in certain cases, the active edge of bone tools remained unmodified, retaining sharpness from their initial use.

The pointed area of the bone used for making initial incisions appears to be a sought-after tool region, and the experimental data validated its effectiveness.

This is the first demonstration of use-wear on bone items from late lower Palaeolithic sites. Both unshaped and shaped bones were effectively used as tools. Fragments of bones, likely byproducts from marrow exploitation, were possibly utilised as a tool if they possessed a sharp and pointed morphology. Moreover, bones were knapped and retouched to produce the desired morphologies to accomplish different tasks.

These data indicate the presence of bone fragments, bone flakes and retouched bones from late lower Palaeolithic sites onwards. These items are sparsely distributed in these sites, suggesting that they were present not due to occasional use or intentional production but due to the primary role of these items in the toolkit, which we attribute to the lithic industry^18,35,36^^and reference there in^.

A study conducted on bone fragments from Isoletta, Colle Avarone and Selvotta (Latium, Italy) demonstrated the ability of hominins to use land and exploit resources during the MIS 11/10 period. The ability to exploit animal carcasses not only as a food source but also in crafting tools reflects the high behavioural flexibility of humans, who exhibited the capacity to interact not only within their social group but also with the broader environment, demonstrating behaviours that indicate significant cognitive development.

The study underscored the remarkable potential of bone tools, particularly in the pointed active edge portions, enabling precise actions such as butchering animal carcasses by making incisions with the tip and cutting meat with the rest of the active edge. At the same time, as demonstrated by experiments, these sharp edges were useful for soil excavation, potentially for gathering nutrients such as tubers or for capturing insects and small animals.

These bone tools provide new evidence of the technological shift recorded for human populations living in Western Europe after the long and strong glacial event MIS 12 and during the long interglacial MIS 11. They also raise questions about the diversity of activities of the human groups living in southern Europe and possible relationships with more favourable climatic conditions that allowed for the acquisition of diverse food resources (ground and soil resources).

This article provides insight into the potential of bone tools recovered from a series of sites situated in central Italy, a region rich in late lower Palaeolithic sites. The presented data confirm that these ancient human groups used an integrated tool kit of lithic and bone tools to guarantee broad exploitation of the environment they encountered. This finding is reinforced by the discovery of bone tools in Asia in Lower and Middle Palaeolithic sites in addition to the tools found in European regions^37 and reference there in^.

## Materials and methods

Use-wear analysis was conducted on a sample of 32 bone items sourced from various portions of long and flat bones found across three sites in southern Lazio. From the Isoletta (Fr) sites, 23 of the 735 items were chosen, while eight of the 640 items were selected from the Colle Avarone (Fr) site, and only one of the 105 items was selected from the Selvotta (Fr) site.

Selection criteria were based on the observable potential of the items, which was evident to the naked eye. We selected items that exhibited a good state of preservation and morphological features indicating potential use (see below). The terms “shaped” and “nonshaped” were used in this article to distinguish between intentionally modified and unmodified tools, respectively.

The analyses were carried out at the Laboratory of Technological and Functional Analyses of Prehistoric Artefacts (LTFAPA) of Sapienza University of Rome.

Various areas on each tool, especially the rounded active edges, were identified. Unfortunately, no evidence of microresidues was found on the surface of the analysed tools.

Prior to analysis, the samples were observed under a stereomicroscope to assess their state of preservation. They were subsequently cleaned with deionised water and neutral Derquim at 2% to remove any surface particles and hand grease that accumulated over time from handling. Each item was then left to air-dry on absorbent paper.

Use-wear analysis involved a low magnification approach (LPA)^38–40^ with a Nikon SMZ-745 stereomicroscope (with 10 × binocular stereomicroscope eyepieces and 1 × objective, magnification ranging between 0. 67 × and 5 × , and a reflected light illumination system) that allowed, through magnifications between 2 and 3 × , identification of the distribution of the traces and the degree of rounding of the active edges. Additionally, a high magnification approach (HPA)^40,41^, was employed with an Optiphot and Nikon Eclipse microscope (with reflected light illumination and 15 × and 10 × eyepieces, 10 × and 20 × lenses and digital ToupCam cameras), which allowed for the identification of the striae and polishes present on the active edges.

Further analysis was conducted using a digital microscope (Hirox RH-2000) with magnifications exceeding 150X and a scanning electron microscope (SEM) (Hitachi TM-3000) to examine the polish texture and stria depth. The analysed samples were subjected to a total vacuum at a voltage of 15 V in analy mode.

Due to the large size of the specimens, a mould of the edge was made using low-grip two-component silicon (Vestige Light Fast) to facilitate insertion under the microscope or into the SEM vacuum room. This process was aimed at preventing surface bone particle detachment. Next, a cast of the mould was made with an epossyc resin (with proportion of Araldite LY554 40% and hardener HY956 10%); the cast was coated with a gold film for SEM analysis.

For the interpretation of use-wear and evaluation of striae—which was not solely based on literature reports—the reference collection available at the LTFAPA laboratory was used, which was supplemented by a dedicated series of experiments involving replicas of bone tools used on materials with different consistencies. Our experiments were conducted on fresh bones of cattle purchased from a butcher shop in Italy that was legally authorised by Italian law to market the meat and bones of cattle from authorised slaughterhouses in compliance with European regulations.

### Supplementary Information

Supplementary Figures.

## Data Availability

All data generated or analysed during this study are included in this published article [and its supplementary information files].
