# Supplementary material for: The use of bones as tools in Late Lower Paleolithic of Central Italy
Source: Sci Rep. 2024 May 22;14:11666. doi: 10.1038/s41598-024-62612-z (PMC11111801; doi:10.1038/s41598-024-62612-z)
Supplement: Supplementary file 1 — Supplementary Figures. [file 41598_2024_62612_MOESM1_ESM.docx]

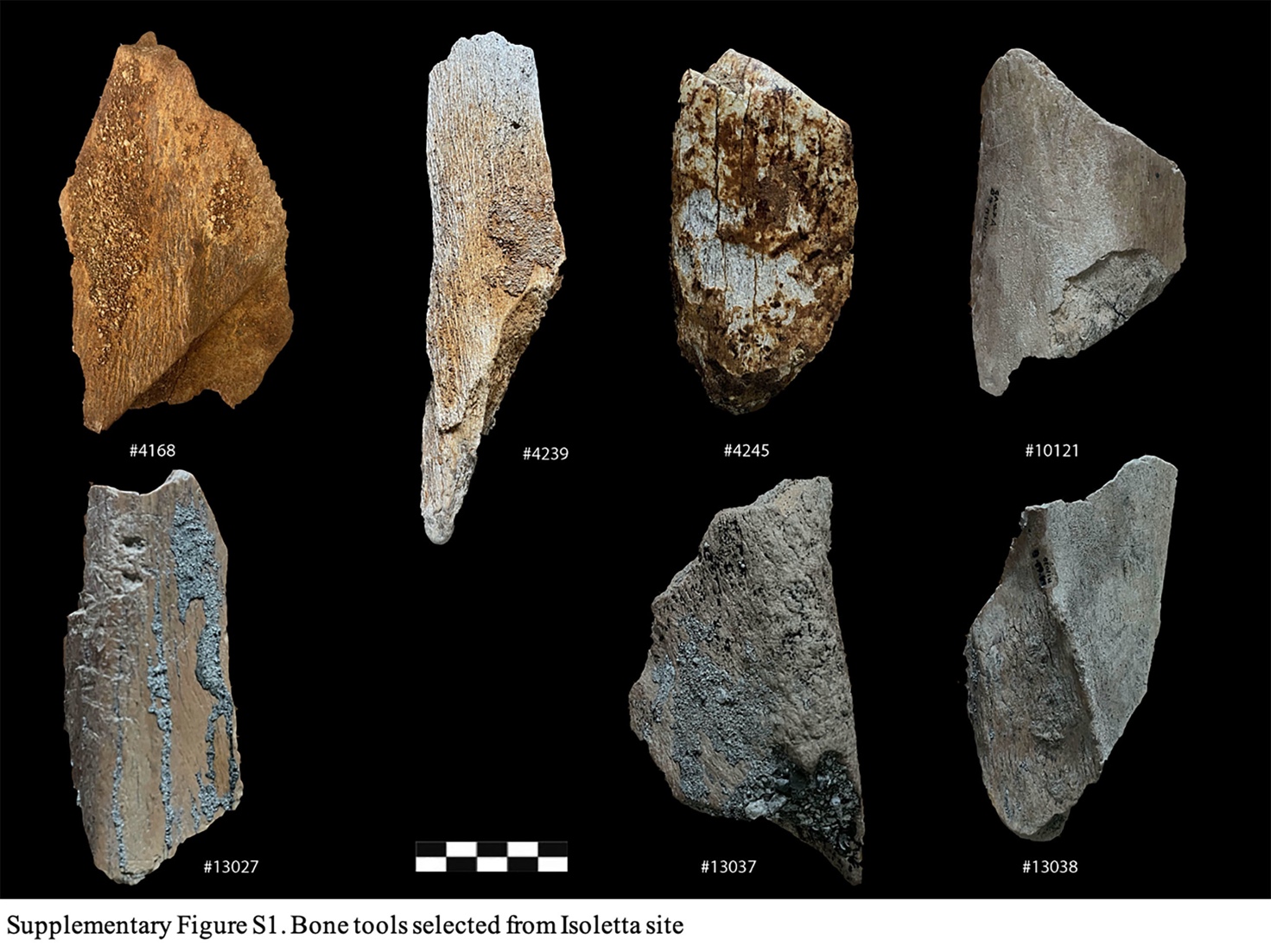


**Supplementary Figure S1. Bone tools.** Sample selected from Isoletta site


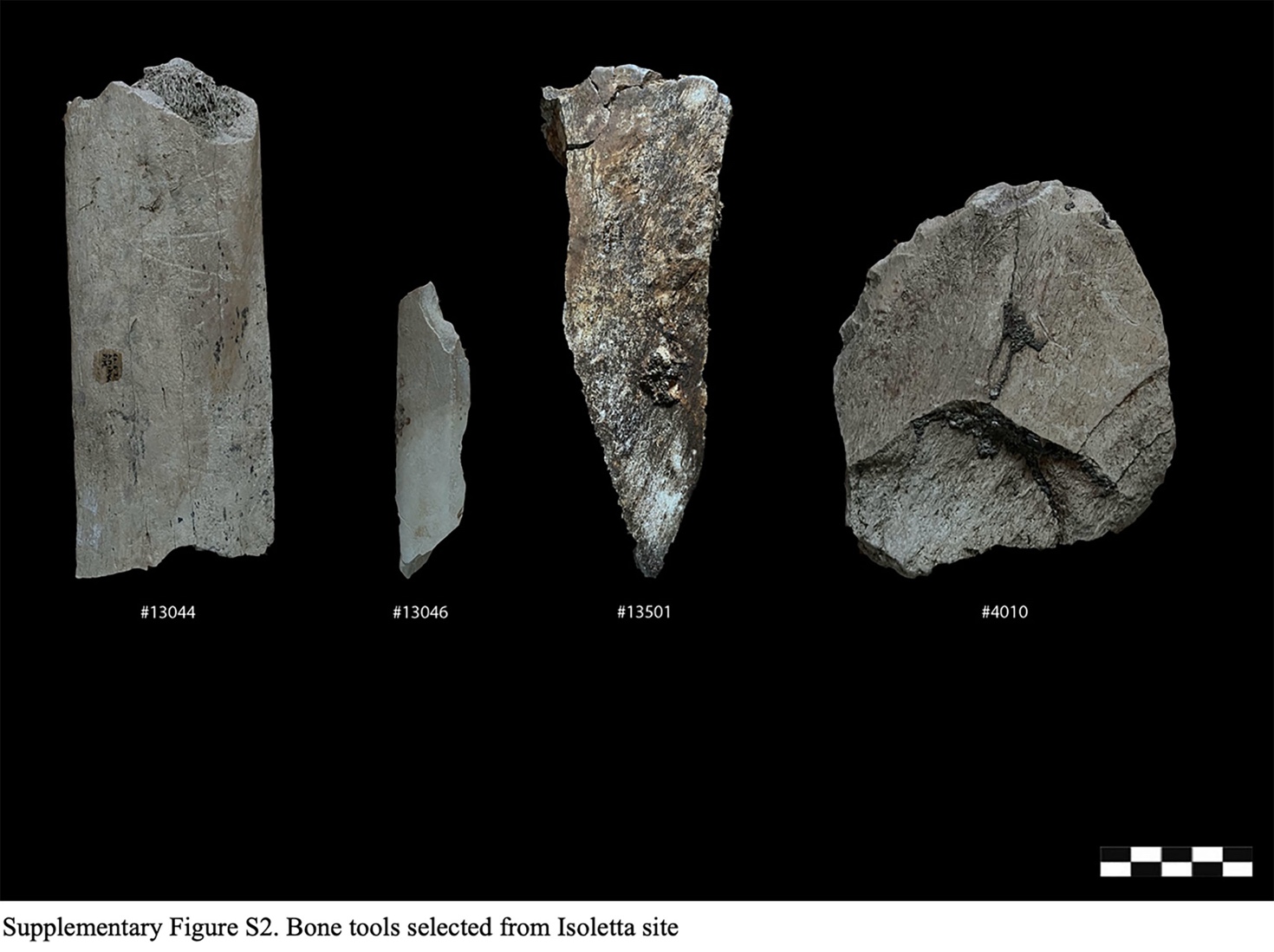


**Supplementary Figure S2. Bone tools.** Sample selected from Isoletta site


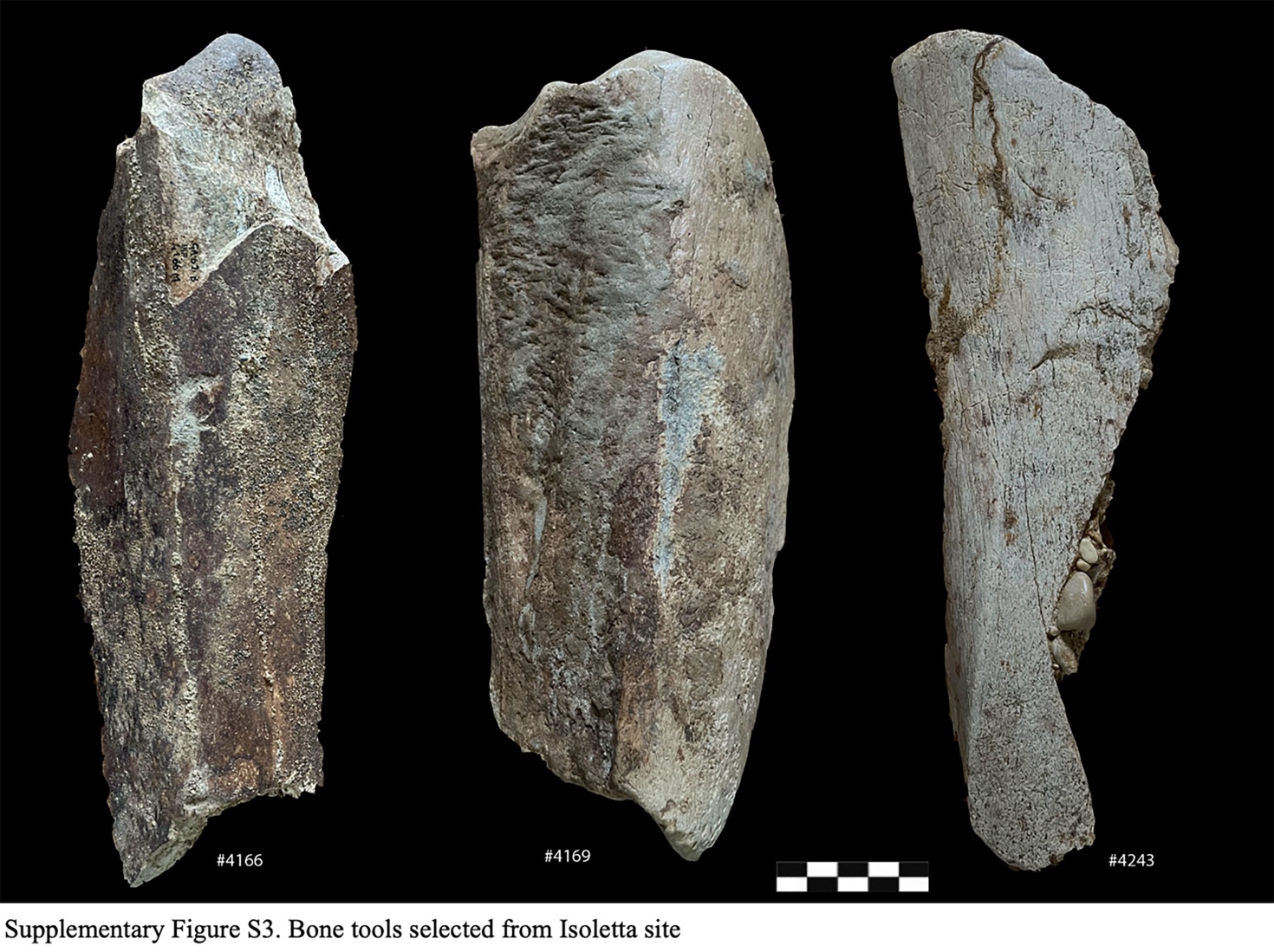


**Supplementary Figure S3. Bone tools.** Sample selected from Isoletta site.


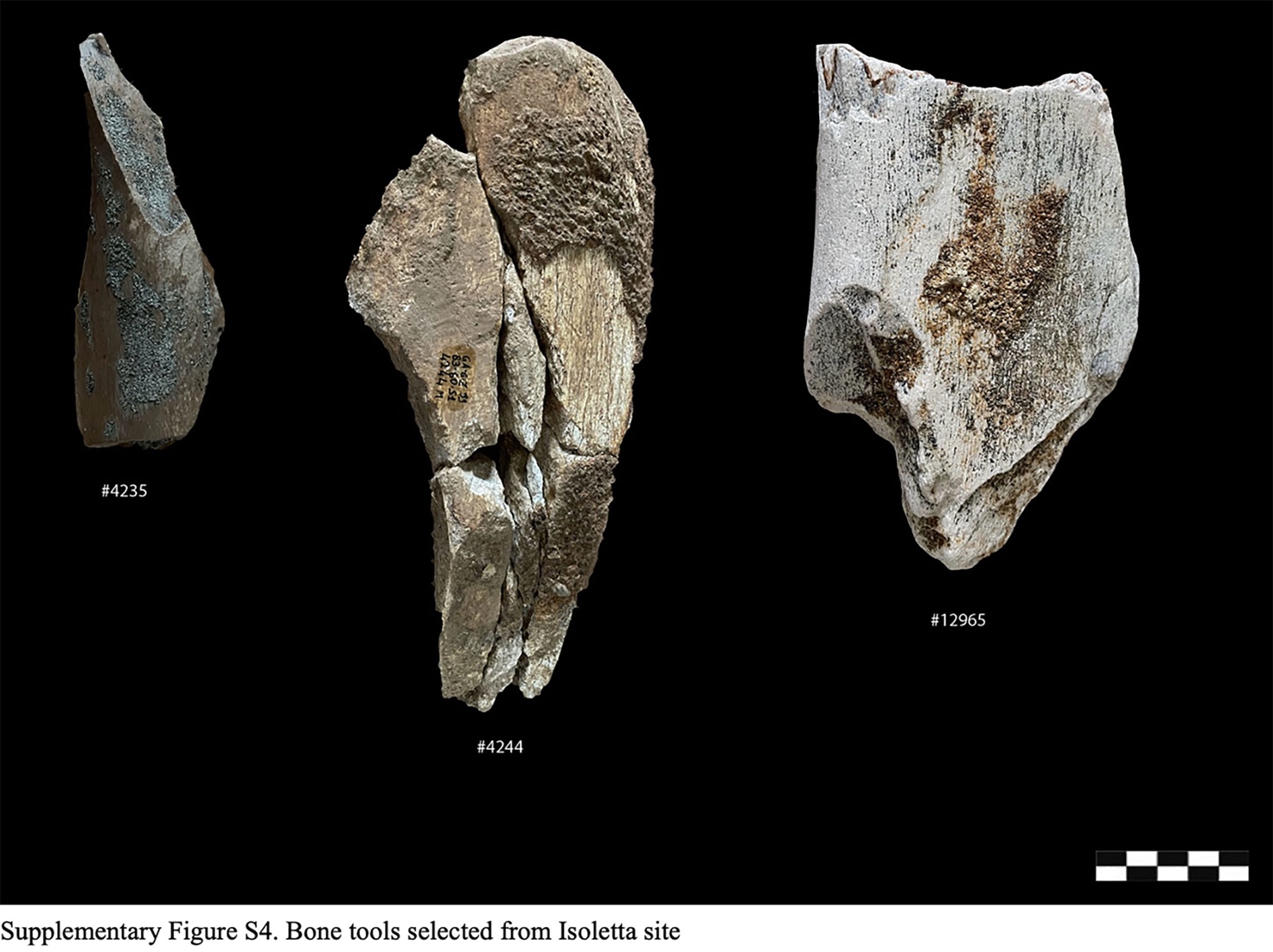


**Supplementary Figure S4. Bone tools.** Sample selected from Isoletta site.


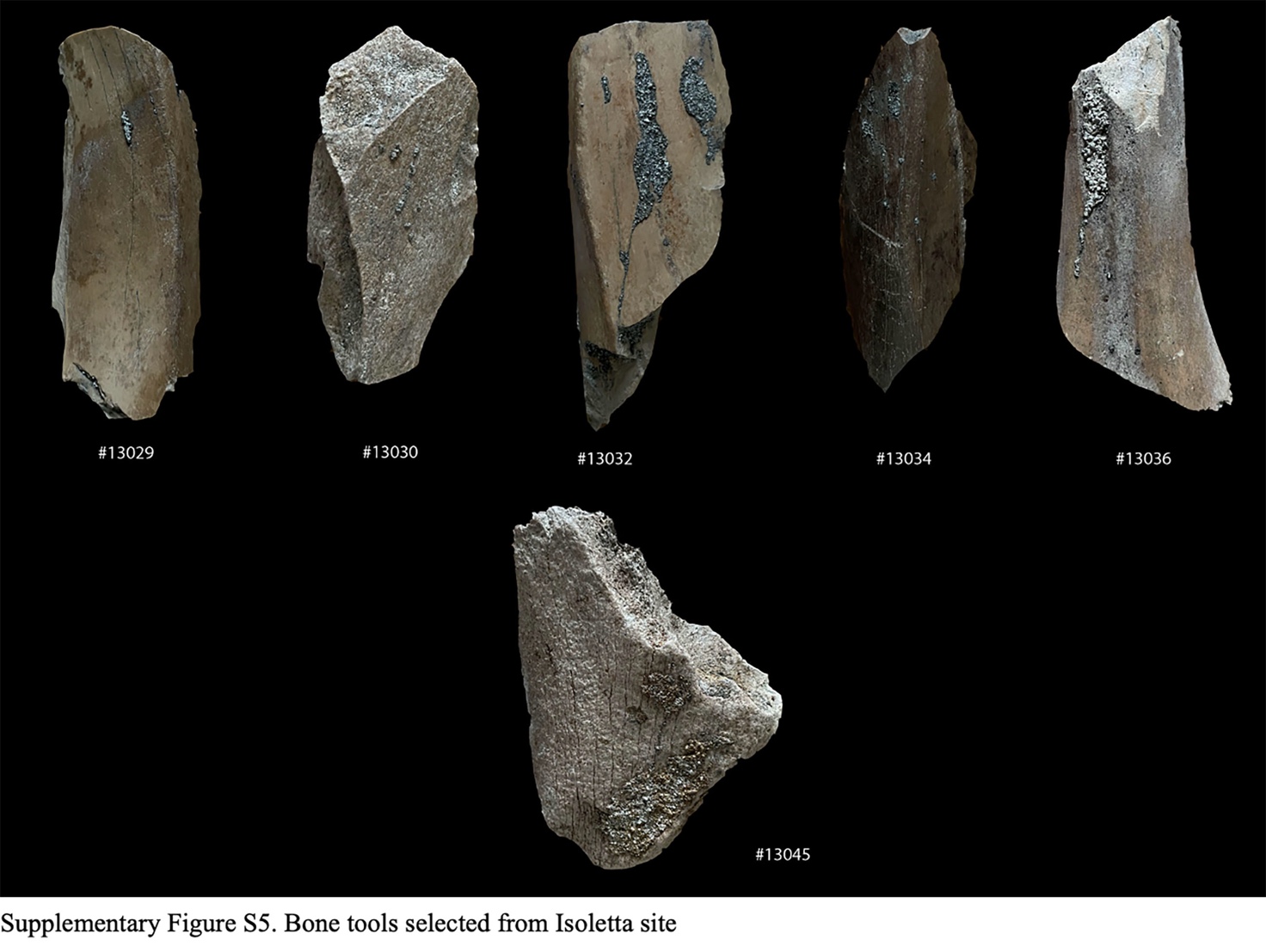


**Supplementary Figure S5. Bone tools.** Sample selected from Isoletta site.


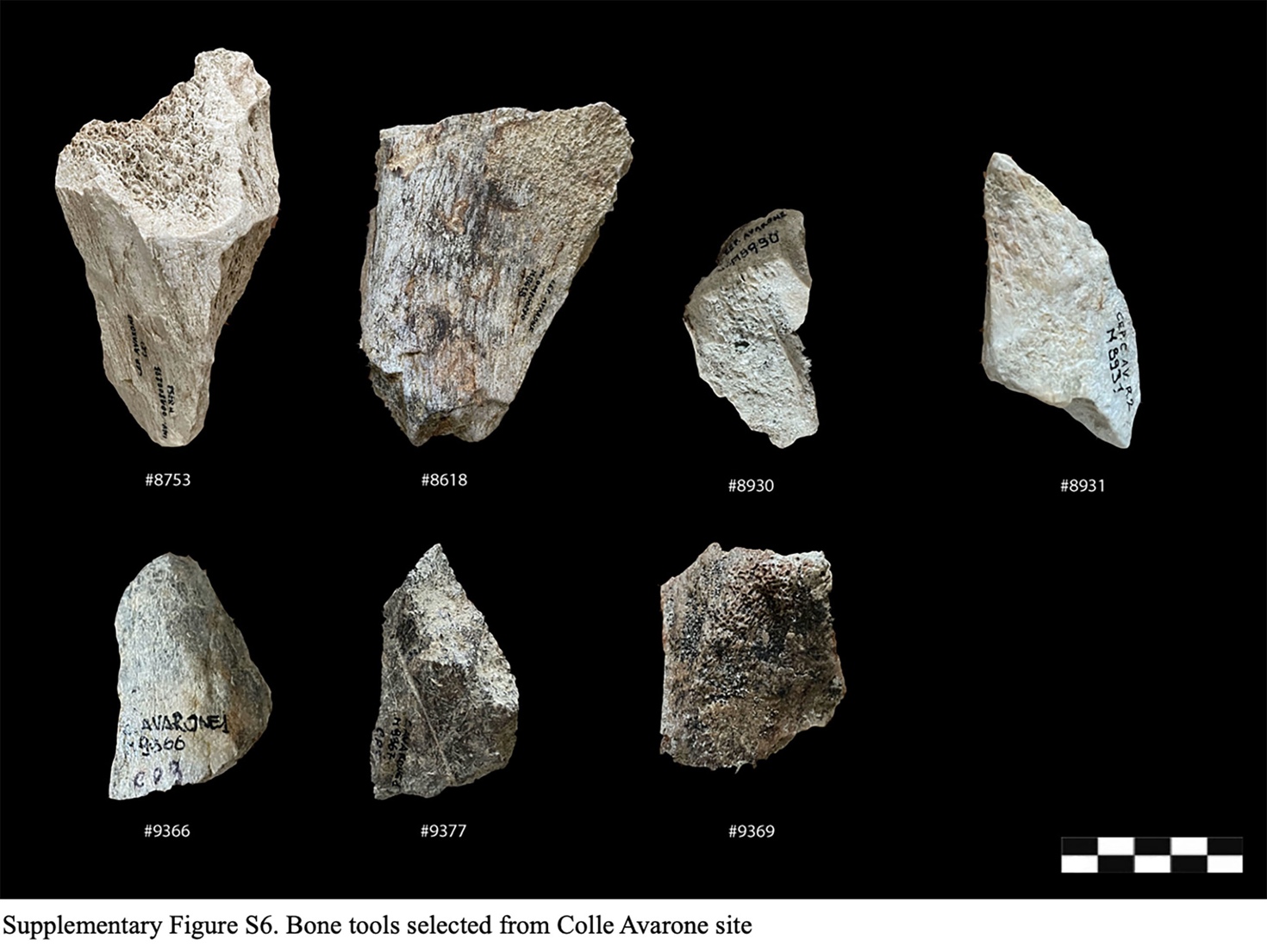


**Supplementary Figure S6. Bone tools.** Sample selected from Colle Avarone site.


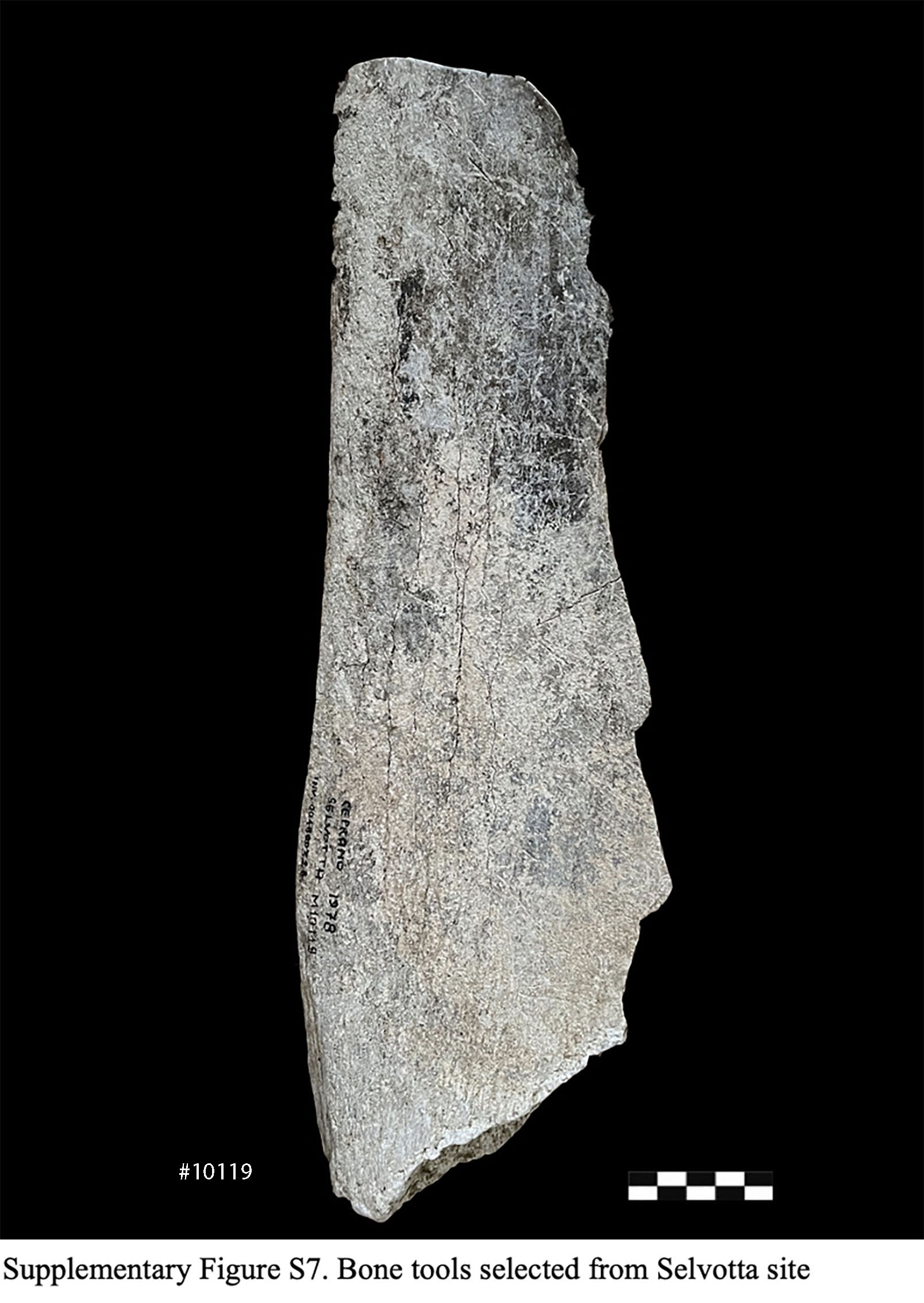


**Supplementary Figure S7. Bone tools.** Sample selected from Selvotta site.


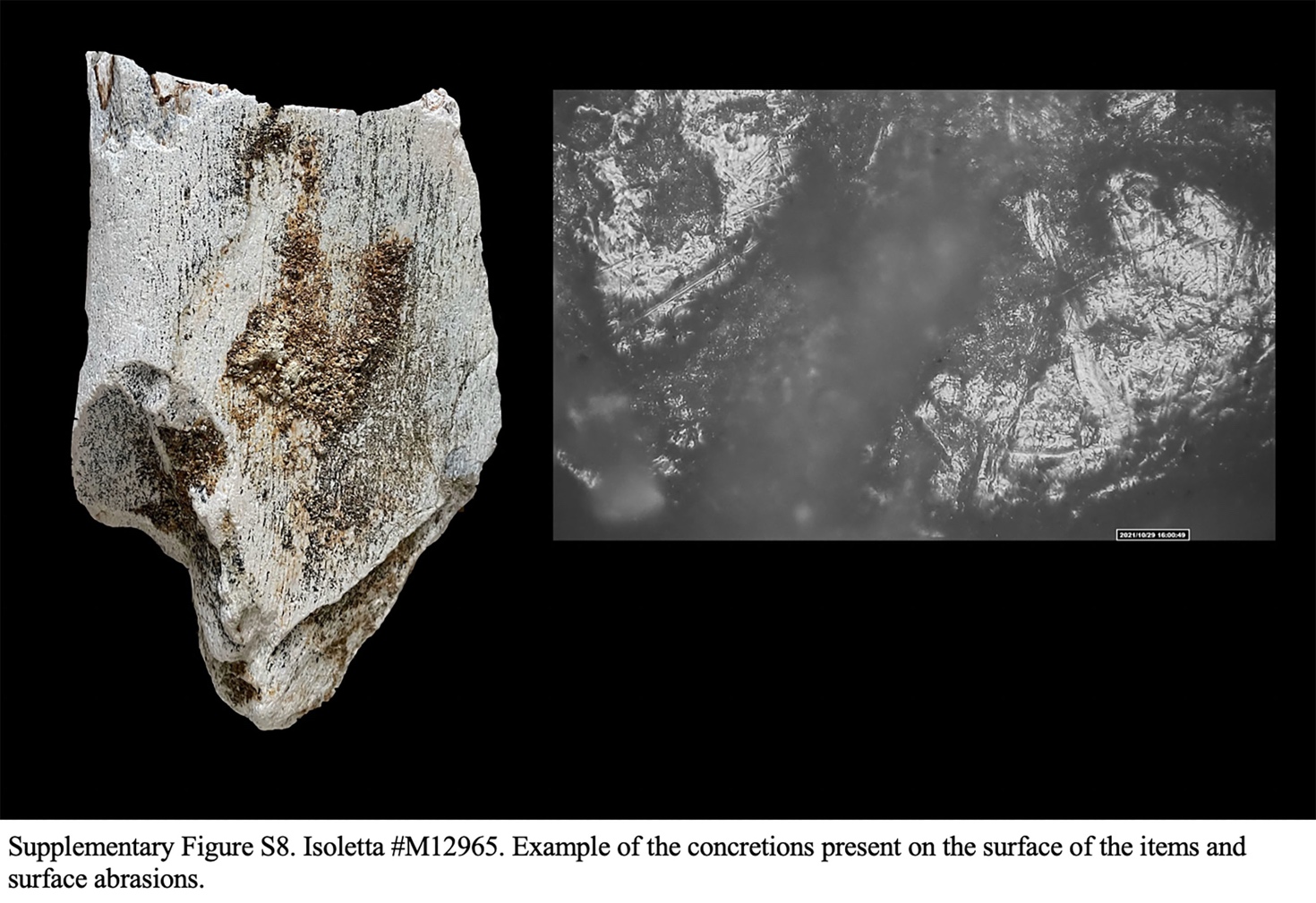


**Supplementary Figure S8. Item #M12965 from Isoletta site**. Example of the concretions present on the surface of the items and surface abrasions.


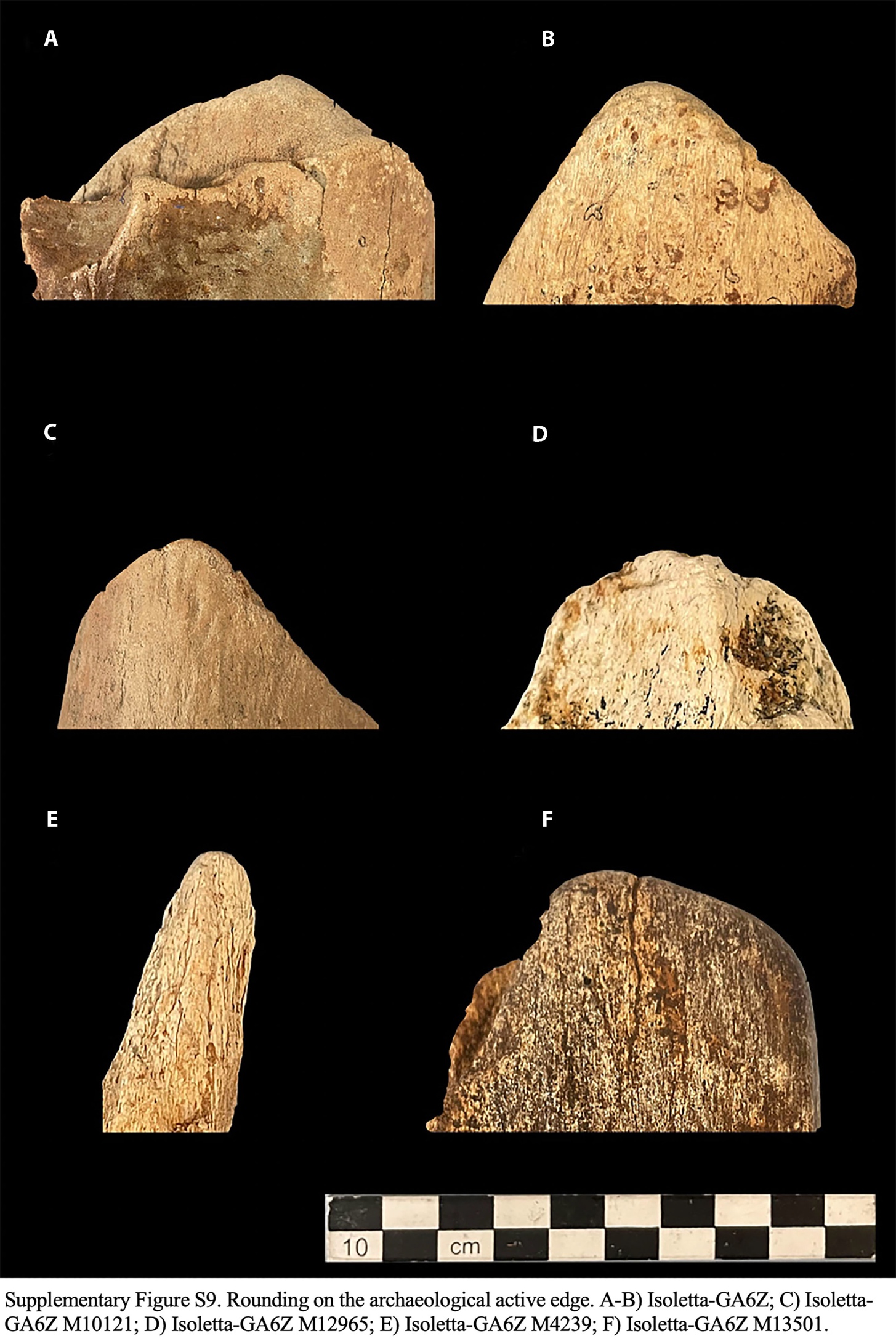


**Supplementary Figure S9. Modifications of the item surface.** A-B) Isoletta-GA6Z; C) Isoletta-GA6Z M10121; D) Isoletta-GA6Z M12965; E) Isoletta GA6Z M4239; F) Isoletta-GA6Z M13501.


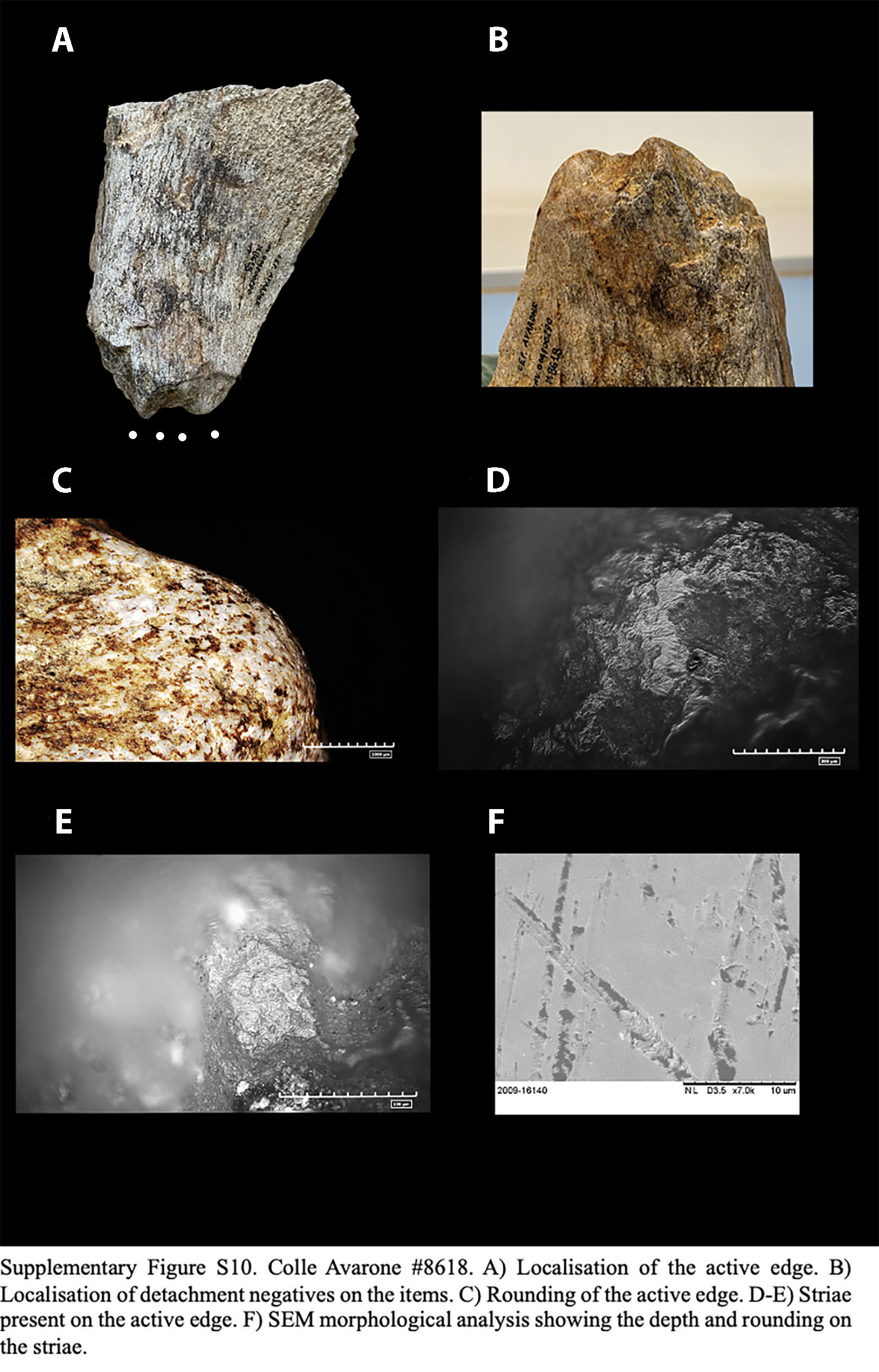


**Supplementary Figure S10. Polish, rounding and stries on a bone tool.** A) Localisation on the active edge. B) Localisation of detachment negatives on the items. C) Rounding of the active edge. D-E) Striae present on the active edge. F) SEM morphological analysis showing the depth and rounding on the striae.


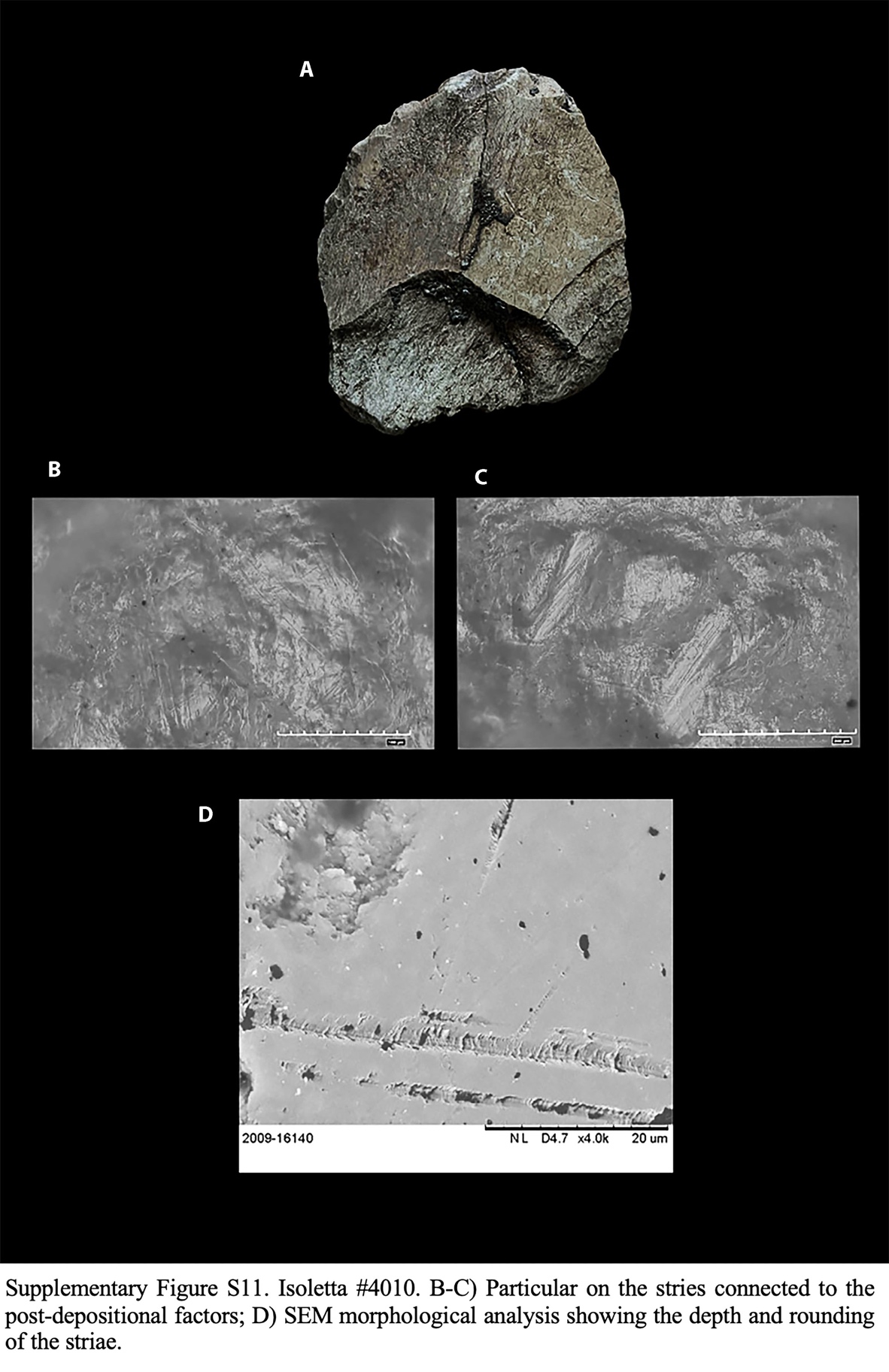


**Supplementary Figure S11. Polish and stries on a bone tool.** Isoletta #4010. B-C) Particular on the stries connected to the post-depositional factors; D) SEM morphological analysis showing the depth and rounding of the striae.


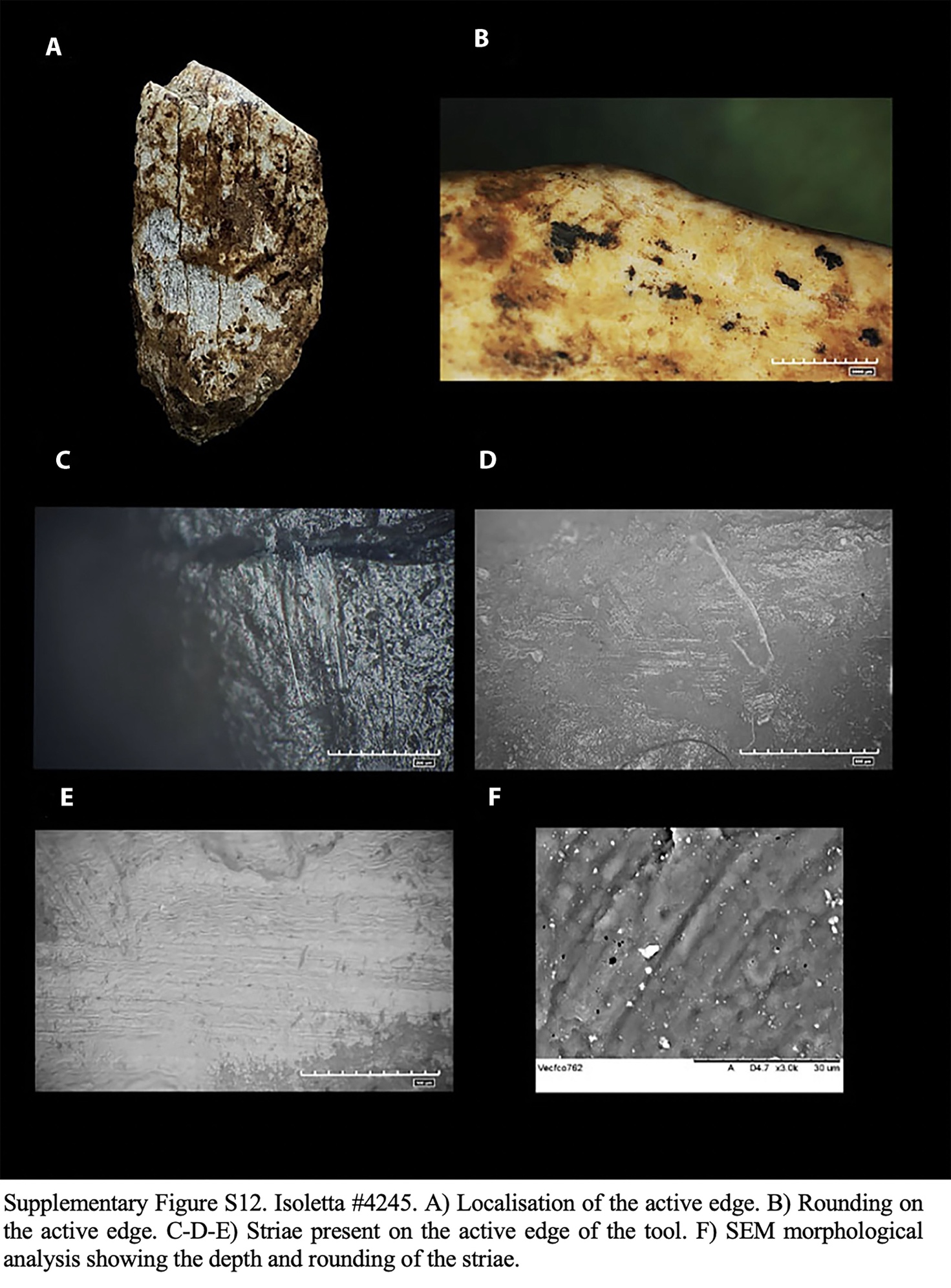


**Supplementary Figure S12. Polish, rounding and stries on a bone tool.** Isoletta #4245. A) Localisation of the active edge. B) Rounding on the active edge. C-D-E) Striae present on the active edge of the tool. F) SEM morphological analysis showing the depth and rounding of the striae.


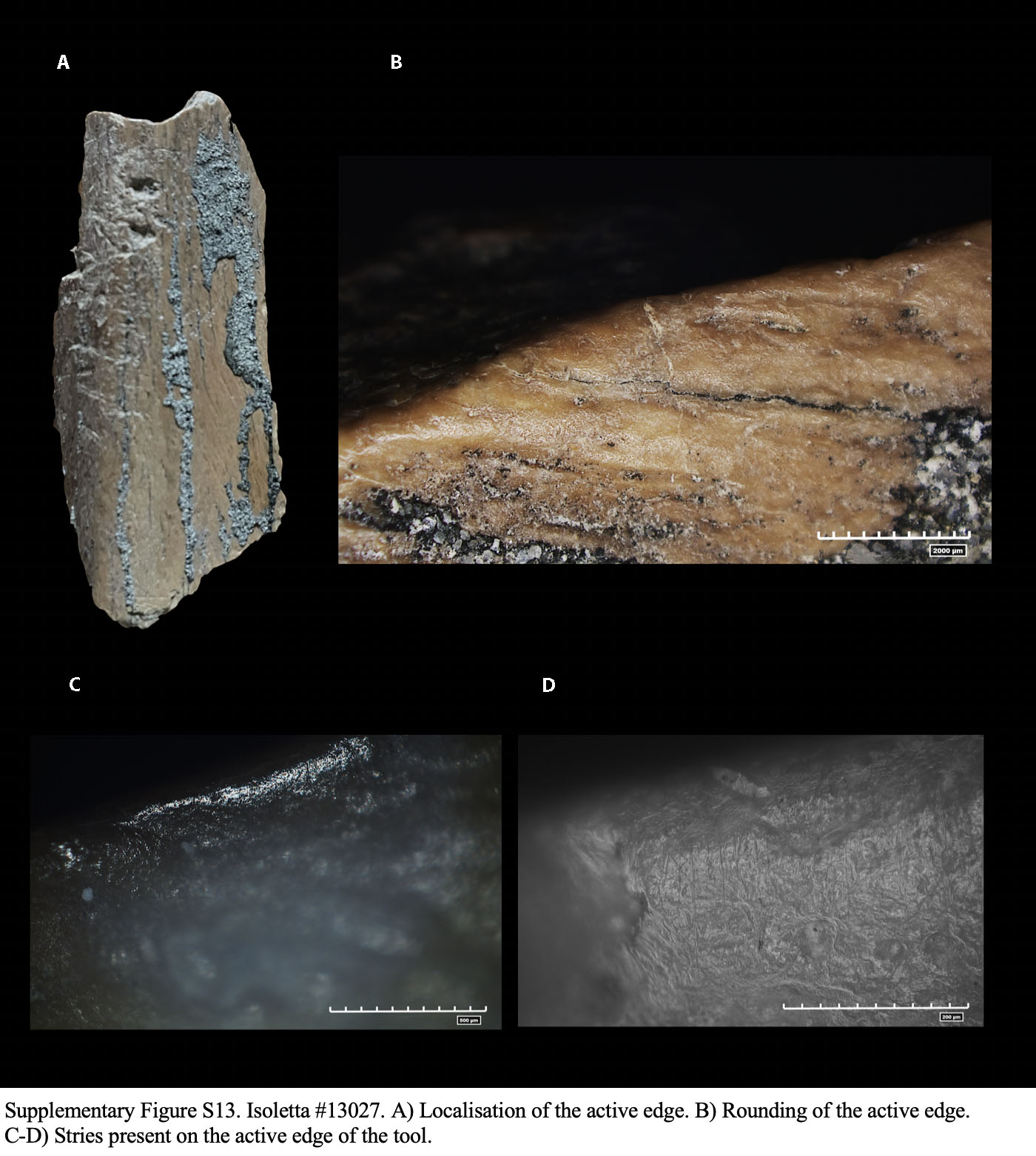


**Supplementary Figure S13. Polish, rounding and stries on a bone tool.** Isoletta #4245. A) Localisation of the active edge. B) Rounding on the active edge. C-D) Striae present on the active edge of the tool.


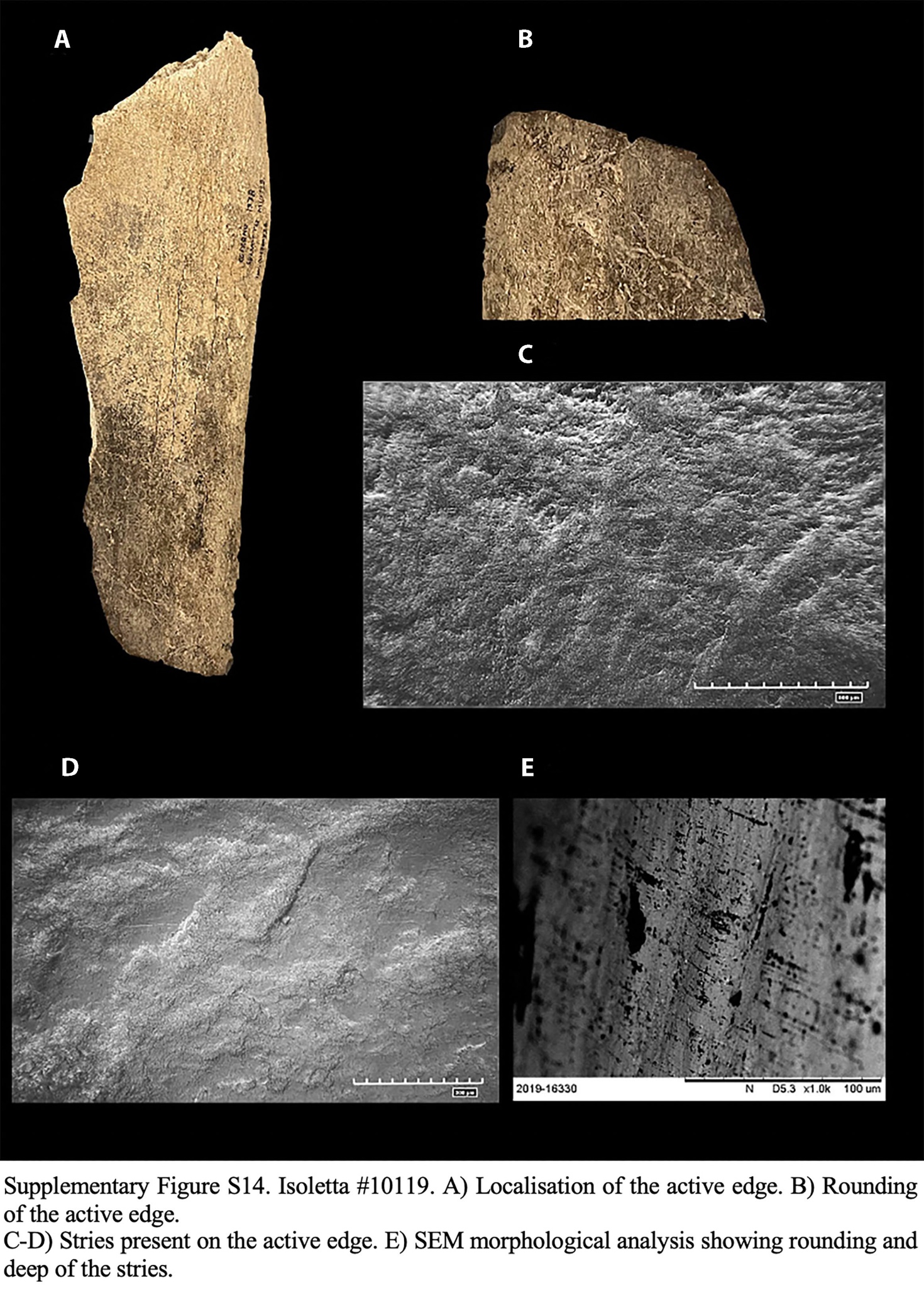


**Supplementary Figure S14. Polish, rounding and stries on a bone tool.** Isoletta #10119. A) Localisation of the active edge. B) Rounding of the active edge. C-D) Striae present on the active edge. E) SEM morphological analysis showing rounding and deep of the stries.
